# En-bloc resection of a giant retroperitoneal lipoma: a case report and review of the literature

**DOI:** 10.1186/s13104-015-1038-7

**Published:** 2015-03-10

**Authors:** Maximilian Weniger, Jan G D’Haese, Wolfgang Kunz, Sebastian Pratschke, Markus Guba, Jens Werner, Martin K Angele

**Affiliations:** Department of General, Visceral, Transplantation, Vascular and Thoracic Surgery, Campus Grosshadern, Ludwig Maximilians-University, Marchioninistraße 15, D-81377 Munich, Germany; Institute for Clinical Radiology, Campus Grosshadern, Ludwig Maximilians-University, Munich, Germany

**Keywords:** Well-differentiated liposarcoma, En-bloc resection, Tumor debulking

## Abstract

**Background:**

Retroperitoneal lipomas are an extremely rare condition with only 17 cases described in the literature since 1980. They can reach enormous size and cause significant abdominal symptoms. The most important differential diagnosis is the well-differentiated liposarcoma, which preoperatively often may not definitely be ruled out.

**Case presentation:**

We present the case of a 73 year-old Caucasian patient with a giant retroperitoneal lipoma of 9 kg measuring 55 cm in diameter. The patient presented with abdominal pain and swelling that had been slowly progressive for the last 15 years. On computerized tomography an immense retroperitoneal tumor was revealed. Intraoperatively, the tumor did not show any signs of infiltrative growth, therefore sole tumor extirpation was performed.

**Conclusion:**

Retroperitoneal lipomas are not clearly distinguishable from well-differentiated liposarcomas on imaging and even biopsies may be misleading. Moreover, abdominal symptoms, i.e. pain, obstipation and dysphagia may occur due to mechanical displacement. Therefore, surgical exploration with complete oncological resection is the therapy of choice if malignity cannot be ruled out.

**Electronic supplementary material:**

The online version of this article (doi:10.1186/s13104-015-1038-7) contains supplementary material, which is available to authorized users.

## Background

Lipomas are benign tumors of mature adipocytes [1] which are commonly located in the subdermal tissue of the trunk and extremities, but rarely retroperitoneally [2,3]. The etiology of lipomas remains unclear. Lately a positive adipocyte stem cell turnover has been supposed as the underlying mechanism [4]. Retroperitoneal lipomas are a rare condition and only 17 cases of retroperitoneal lipomas in adults have been described in the literature since 1980 [5-20] (Table 1). Here we present the case of a 73 year-old female with a giant retroperitoneal lipoma filling great parts of the abdomen and measuring 55 x 40x 10 cm. While retroperitoneal lipomas are rare by themselves, only few retroperitoneal lipomas of greater size have been reported in the literature.

**Table 1:** **Summary of all case reports describing retroperitoneal lipomas resected in adults since 1980**

	**Age**	**Sex**	**Tumor size**	**Weight**
Saito S. *et al*., 2013 [5]	65	male	30 cm in diameter	No data
Wei D. *et al*., 2013 [6]	25	female	20 x 12 x 10 cm	1650 g
Chander *et al*., 2012 [7]	36	female	13,6 x 11,2 x 9,1 cm	1300 g
Singh G. *et al*., 2011 [8]	65	male	25 x 12 cm	No data
Ukita S. *et al*., 2009 [9]	61	female	15 cm in diameter	no data
Ida C. *et al*., 2008 [10]	65	male	22 x 14 x 5 cm	no data
Drop A. *et al*., 2003 [11]	72	female	12 x 9 x 4 cm	no data
Drop A. *et al*., 2003 [11]	60	female	13 x 12 cm	no data
Martinez C. *et al*., 2003 [12]	32	female	20 x 13 x 10 cm	3400 g
Raftopoulos I. *et al*., 2002 [13]	62	male	20 x 15 x 10 cm	790 g
Foa C. *et al*., 2002 [14]	52	male	10,5 x 9,5 x 2 cm	145 g
Forte *et al*., 2002 [15]	61	male	no data	no data
Marshall M. *et al*., 2001 [16]	47	male	no data	4990 g
Matsubara N. *et al*., 2000 [17]	65	male	12 x 13 cm	no data
Acheson A. *et al*., 1997 [18]	76	female	20 x 20 x 12 cm	596 g
Zhang S. *et al*., 1987 [19]	65	male	50 cm in diameter	19.5 kg
Deppe G. *et al*., 1985 [20]	26	female	11 x 8 x 3 cm	no data

## Case presentation

**Figure 1:**
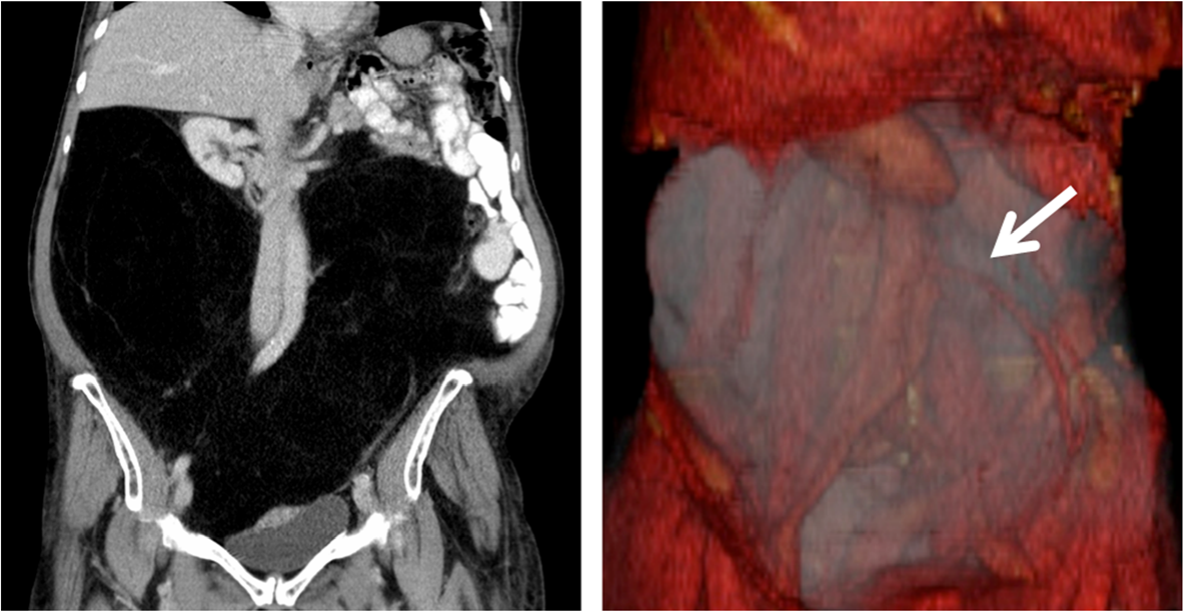
**The tumor on computerized tomography and on 3D-reconstruction.** On the left the tumor is displayed on coronal plane, showing massive shifting of the intestines and kidneys. On the right the tumor is shown on 3D-reconstruction, nearly filling the whole abdomen with encasement of the inferior mesenteric artery (white arrow).

A 73 year-old, Caucasian female was referred to our center with a giant retroperitoneal mass. The patient complained about significant abdominal swelling and recurrent episodes of abdominal pain and obstipation. The tumor had been slowly progressive over the past 15 years. On physical examination the patient’s abdomen was greatly distended and non-tender on palpation. Blood tests showed no significant pathologies. A computerized tomography (CT) scan showed the giant retroperitoneal mass (Figure 1). CT-guided core biopsy samples of this mass demonstrated histology of a benign lipoma with no signs of malignancy. Due to the massive size the mass was considered as radiologically highly suspect for low grade liposarcoma. After interdisciplinary discussion of this case in our sarcoma tumor board, exploratory laparotomy was indicated with the aim of complete tumor resection for potential malignancy or at least tumor debulking to reduce abdominal symptoms.

**Figure 2:**
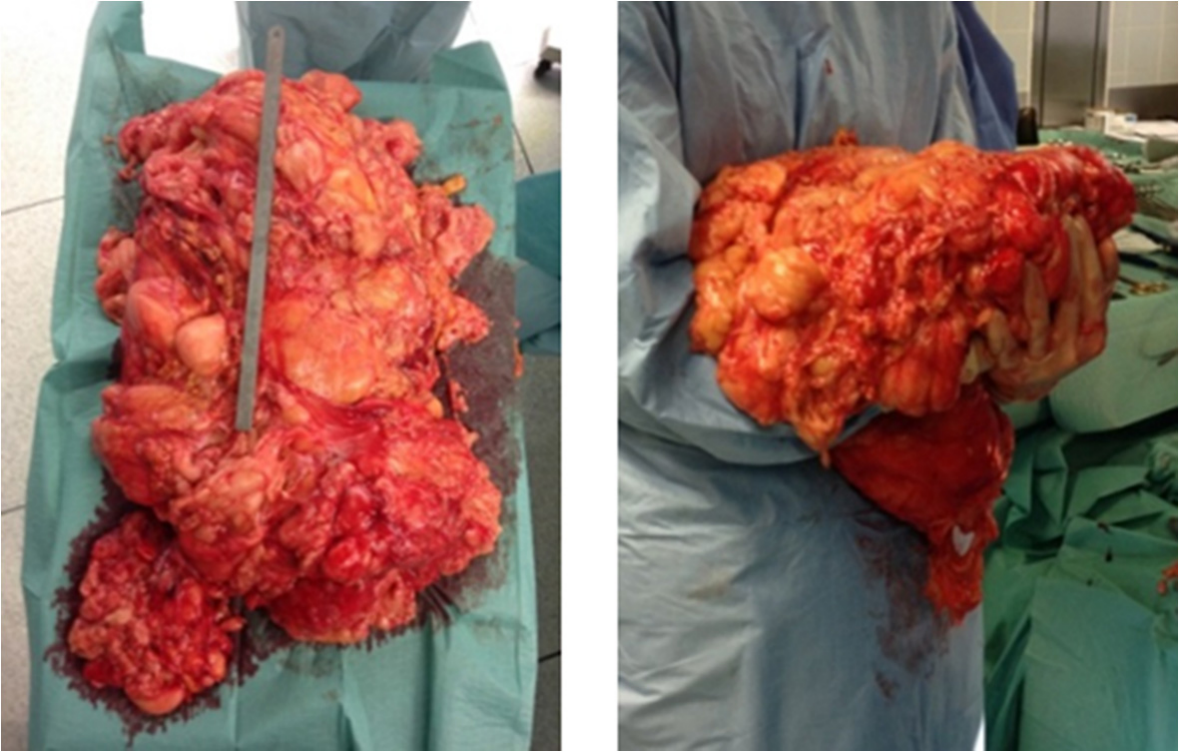
**The tumor shortly after en bloc-resection.** On the left the tumor is shown with a metric tape measure of 30 cm. On the right the tumor is being held by one of the operators shortly after en-bloc resection.

Intraoperatively a giant, clearly demarcated fatty tumor adherent to the right retroperitoneal fatty tissue measuring 55 x 40 x 10 cm was evident (Figure 2). Although the tumor was distending the mesenteric artery the mass was completely extirpated without resection of adjacent tissue or organs. The patient’s postoperative course was complicated due to prolonged paralytic ileus and intestinal distention, which was successfully managed conservatively. The patient was discharged on the 18th postoperative day from the hospital. The pathologic specimen showed a lipomatous tumor weighing 8.95 kg with mature adipocytes and without signs of nuclear atypia. The staining for MDM2 (MDM2 proto-oncogene) and CDK4 (cyclin-dependent kinase 4) was negative, therefore the lesion was diagnosed as a lipoma.

## Discussion

Giant retroperitoneal lipomas in adults are scarce, with a total number of 17 cases described in the literature since 1980 [5-20] (Table 1). Subcutaneous lipomas are associated with hypercholesterolemia [21,22], obesity [23-25] and trauma [26], whereas such data does not exist for retroperitoneal lipomas. Moreover, the patient was not described positive for any of these factors. [27]. Retroperitoneal lipomas are usually asymptomatic for a long time before they cause abdominal swelling or symptoms due to obstruction or shifting of adjacent organs and structures. At this point, they may have already reached enormous size. This may be explained by the great retroperitoneal space that allows them to grow before they get symptomatic and the slowness of their enlargement. The diagnosis is based on a MRI (magnetic resonance imaging) or CT-scan, yet both imaging modalities may not exclude a well-differentiated liposarcoma. Furthermore, biopsies often remain inconclusive. The incidence of soft-tissue sarcomas in general is described with 4 cases per 100.000 and year [28], of which liposarcomas are the most common and are located in one third of cases in the retroperitoneum [29]. The incidence of retroperitoneal lipomas on the other hand is thought to be extremely rare. Hence, a well-differentiated liposarcoma may not be ruled out preoperatively, even if a benign lipoma is clinically suspected. Therefore, intraoperative judgment about tumor characteristics and subsequent decision making about the extent of resection is of great importance. Resection with negative margins (R0) is crucial to the patient’s prognosis in case of a liposarcoma, therefore a wide excision should be carried out if infiltrative growth is suspected or if there is any doubt about dignity [30]. Sole extirpation should be reserved for clearly circumscribed tumors. However, due to the enormous size of the tumor preoperative judgment about resectability based on CT-scans is difficult. Therefore, tumor debulking for symptom relief can also be discussed if oncological resection is not feasible. Due to the possible malignant nature of such retroperitoneal tumors resection should be carried out by a trained oncological surgeon in a center of excellence for soft-tissue sarcomas [30]. In this case the tumor was clearly demarcated macroscopically and without any sign of infiltrative growth. Moreover, the enormous size did not allow accomplishing an oncological correct resection. Thus, sole extirpation was performed. Since clinical and experimental data on tumor progression and tumor recurrence of retroperitoneal lipomas are lacking a close and regular follow-up is indicated.

## Conclusion

Retroperitoneal lipomas are a rare condition. The presented case points out that abdominal lipomas can grow to enormous size and cause clinically significant symptoms and, even if massively enlarged, are resectable with good clinical outcome. Further research is required to fully understand the underlying etiology and genetic mechanisms. The most important differential diagnosis is the more frequent well-differentiated liposarcoma, therefore oncological resection should always be considered.

## Consent

Written informed consent was obtained from the patient for publication of this Case Report and any accompanying images. A copy of the written consent is available for review by the Editor-in-Chief of this journal.

## Abbreviations

CT: Computerized tomography
MDM2: MDM2 proto-oncogene
CDK4: Cyclin-dependent kinase 4
MRI: Magnetic resonance imaging
